# The Inflammatory Response to Ruptured Abdominal Aortic Aneurysm Is Altered by Endovascular Repair

**DOI:** 10.1155/2013/482728

**Published:** 2013-12-02

**Authors:** R. R. Makar, S. A. Badger, M. E. O'Donnell, C. V. Soong, L. L. Lau, I. S. Young, R. J. Hannon, B. Lee

**Affiliations:** Vascular and Endovascular Surgery Unit, Belfast City Hospital, Lisburn Road, Belfast BT9 7AB, UK

## Abstract

*Introduction*. Ruptured abdominal aortic aneurysm (rAAA) causes a significant inflammatory response. The study aims to investigate this response following endovascular and open repair of ruptured AAA. *Patients and Methods.* Consecutive rAAA patients had either endovascular aneurysm repair (EVAR) or open repair (OR). Blood samples were taken for cytokines, lipid hydroperoxides (LOOH), antioxidants, and neutrophil elastase/**α**1-anti-trypsin complexes (NE/AAT) before surgery, 6 hours after clamp release and 1, 3, 5 days postoperatively. *Results*. 30 patients were included in the study, with 14 undergoing eEVAR and 16 eOR, with comparable baseline comorbidities, age, and parameters. IL-6 peaked higher in eOR patients (*P* = 0.04), while p75TNFr was similar between groups except at day 5 (*P* = 0.04). The NE/AAT concentrations were higher in eOR patients (*P* = 0.01), particularly in the first postoperative day, and correlated with blood (*r* = 0.398, *P* = 0.029) and platelet (*r* = 0.424, *P* = 0.020) volume transfused. C-reactive protein rose and lipid hydroperoxide fell in both groups without significant intergroup difference. Vitamins C and E, lycopene, and **β**-carotene levels were similar between groups. *Conclusion*. EVAR is associated with lower systemic inflammatory response compared to OR. Its increased future use may thereby improve outcomes for patients.

## 1. Introduction

Ischaemia-reperfusion injury plays an important role in the development of multiorgan failure following abdominal aortic aneurysm (AAA) repair. Shock in ruptured aneurysm (rAAA) results in global ischemia, and reestablishing the blood supply can cause further tissue injury due to the generation of oxygen-derived free radicals [1]. Normally the reactive oxygen species (ROS) are produced in small amounts via aerobic metabolic pathways, with a fine balance between the prooxidants and antioxidants. ROS damage cellular molecules leading to DNA fragmentation, membrane damage and lipid peroxidation, and consequently to cell death [2, 3].

Lipid peroxidation produces lipid hydroperoxide (LOOH), thus damaging the vascular endothelium, with increased capillary permeability to protein and consequently tissue oedema and renal albumin excretion [4, 5]. Activation of the arachidonic acid cascade leads to the generation of thromboxanes, prostaglandins, and leukotrienes. These are potent platelet and neutrophil activators, and vasoconstrictors [6, 7]. Neutrophil-dependent injury, important in the genesis of SIRS and MODS, is characterised by the adhesion of activated polymorphonuclear neutrophils (PMNs) to endothelium and their release of metabolites, resulting in capillary leakage [8].

Neutrophil elastase is associated with lung injury and ARDS [9]. Fujishima et al. reported significantly increased plasma levels of neutrophil elastase/*α*1-antitrypsin complex in patients with ARDS [10]. Tanaka et al. observed an increase in plasma neutrophil elastase/*α*1-antitrypsin complex in patients with both septic and haemorrhagic shock [9]. Increasing neutrophil elastase/*α*1-antitrypsin complex has been observed in patients undergoing open abdominal aortic aneurysm repair [11].

The nonenzyme defenses include antioxidant vitamins (C, E, and carotenoids), free metals, and haem binding proteins. Low plasma antioxidants concentrations are associated with MODS and poor outcome in critically ill patients [12]. Reductions in antioxidant concentrations following open AAA repair are controversial [13]. Vitamin C protects against cell membrane lipid peroxidation by potentiating the activity of tocopherol [14]. Carotenoids are a group of over 500 fat-soluble pigments such as *β*-carotene, *α*-carotene, and lycopene, the latter of which is the most efficient [15].

The aim of this prospective nonrandomised study was to investigate the systemic inflammatory response following endovascular and open repair of ruptured AAA.

## 2. Patients and Methods

### 2.1. Patient Selection

The details of selection have been outlined before [16]. However, in brief, patients over the age of 50 years presenting to the Vascular Surgery Unit of the Belfast City Hospital with a diagnosis of infrarenal ruptured AAA were recruited after obtaining consent. All patients underwent preoperative CT scan to assess suitability for endovascular repair. Otherwise, an open surgical technique was used. Haemodynamic stability was not a factor to determine operative strategy. In the presence of instability, an aortic occlusion balloon was used. Patients were excluded if they had no evidence of extravasation of blood from the aneurysm sac on CT scan, juxtarenal aortic aneurysm, had dementia, refused to participate, or had chronic renal failure on haemodialysis. Patients who died within 2 hours of repair were also excluded from further analysis.

### 2.2. Blood Samples

Blood samples for cytokine, LOOH, antioxidants, and NE/AAT measurements were taken before surgery, 6 h after clamp release or reperfusion of both lower limbs and then at D1, D3, and D5. The plasma of these blood samples was isolated by centrifugation and immediately stored at −80°C until analysed for IL-6, p75TNFr, NE/AAT complex and vitamins C. In addition, 100 *μ*L of plasma were mixed with 900 *μ*L of 5% metaphosphoric acid (MPA) (Sigma Chemical, Dorset, UK) to stabilise vitamin C prior to sample storage. Another 4 mls were collected for lipid hydroperoxides and antioxidants vitamin E and carotenoids. All samples were assayed in duplicates.

### 2.3. Interleukin-6 (IL-6)

IL-6 was measured in plasma using high-sensitivity quantitative enzyme-linked immunoassay (ELISA). The Quantikine HS kits (SS600B, R&D System Inc, Minneapolis, USA) used in this analysis has 96-well microplates coated with a mouse monoclonal antibody against IL-6. The optical density of each well was determined by using the microplate reader set at 620 nm. The IL-6 plasma concentration was calculated automatically by using the average of the two absorbance values for each sample and the four parameter logistic (4PL) curve-fit. The standard curve was generated for each set of samples assayed by using the six dilutions of standard stock solution in addition to the zero and high standard concentrations. Duplicate samples with variation greater than 10% were repeated.

### 2.4. p75TNFr

p75TNFr was measured by a quantitative sandwich ELIZA using Quantikine human sTNF RII/TNFRSF1B immunoassay kits (DRT200, R&D Systems, Inc. Minneapolis, MN 55413, USA). The 96 wells of the polystyrene microplate were coated with mouse monoclonal antibody specific for p75TNFr. The optical density of each well was determinate by using the microplate reader set at 620 nm. The standard curves were generated for each set of samples assayed by the use of the six dilutions of the standard stock solution in addition to the zero and high-standard concentrations. Duplicate samples with a variation greater than 10% were repeated.

### 2.5. Neutrophil Elastase/*α*1-Anti-Trypsin Complexes (NE/AAT)

The NE/AAT complex was used as a marker of PMN activity. Plasma NE/AAT complex was measured by using commercially available NE/AAT complex ELIZA kit (QIA96, Calbiochem, EMD Bioscience, Inc, Darmstadt, Germany). This kit consisted of 96 wells coated with a polyclonal antibody (egg yolk) to human neutrophil elastase. The absorbance of each well was read on spectrophotometer at 450 nm. Duplicated samples with variation greater than 10% were repeated.

### 2.6. Lipid Hydroperoxides, *α*-Tocopherol, *β*-Carotene and Lycopene Assays

Serum hydroperoxide concentration was measured in the aqueous phase using Ferric Oxidation of Xylenol Orange: version 1 (FOX1) spectrophotometric method, which is extremely sensitive. The hydroperoxides oxidise ferrous ions (Fe^2+^) to ferric ions (Fe^3+^) selectively in dilute acid solutions. The absorbance was read at 560 nm against the H_2_O_2_ standard curve. Serum *α*-tocopherol, *β*-carotene, and lycopene concentrations were determined by reverse-phase high-performance liquid chromatography (HPLC).

### 2.7. Vitamin C Assay

The vitamin C assay is based on the enzymatic oxidation of ascorbic acid by ascorbate oxidase (Sigma Chemical, Dorset, UK) and subsequent quinoxaline formation with 1,2 phenylenediamine (Sigma Chemical, Dorset, UK) to generate a fluorescent derivative measured on the Cobas Fara II centrifugal analyser (Roche Diagnostic System, Indianapolis, IN, USA).

### 2.8. Statistical Analysis

Data were analysed on SPSS version 15 (SPSS Inc, Chicago, IL, USA). Continuous variables were expressed using mean (±SD) or median (IQR) as appropriate. Intergroup differences were determined using Mann-Whitney *U* test and Wilcoxon Signed Rank test. Correlation was assessed using the Spearman's Rank Correlation Coefficient test. A *P* value of <0.05 was considered statistically significant.

## 3. Results

### 3.1. Patient Characteristics

These results have been reported before [16]. However, in brief, during the two-year study period, 40 consecutive rAAA patients who reached the hospital alive were considered for the study. A total of 10 patients were excluded from the study. Thirty patients were included in the study. Fourteen patients had eEVAR and sixteen underwent an eOR. The average age for the eEVAR group was 72.2 (±6.2) years and the eOR was 71.4 (±7.0) years (Table 1). The male-to-female ratio was 6 : 1 in the eEVAR group and 7 : 1 in the eOR group. The baseline parameters were comparable for the two groups. The eEVAR group was associated with lower blood loss (*P* < 0.001), less blood transfusion (*P* < 0.001), and less total intraoperative intravenous fluid infusion (*P* = 0.001) [16]. Interestingly, the duration of surgery was not significantly different (*P* = 0.34) between the two surgical strategies, but while all eOR patients were admitted to intensive care unit, only 8 of the eEVAR patients did so [16].

### 3.2. IL-6

**Figure 1:**
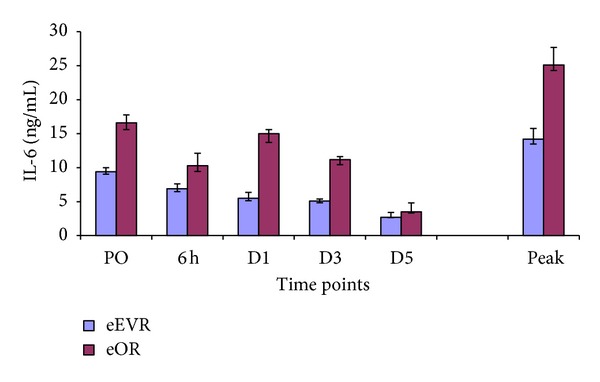
Plasma IL-6 levels expressed as median and interquartile range. At peak *P* = 0.04 eEVR versus eOR; 6 h and D5 versus PO in eOE *P* < 0.05; D3 versus PO in eEVR *P* < 0.05.

IL-6 decreased in both groups after surgery, reaching parity on D5. In the eEVAR group, it was significantly lower on D3 (*P* = 0.03) compared to PO, and in the eOR group the concentrations at 6 h (*P* = 0.02) and D5 (*P* = 0.04) were significantly lower than PO (Figure 1). The peak IL-6 was significantly higher in the eOR group (25.1 (17.2–50.9 pg/mL)) compared to the eEVAR group (1.42 (7.1–29.8 pg/mL; *P* = 0.04)). However, IL-6 levels were similar at the individual time points in both groups.

### 3.3. p75TNFr Assay

**Figure 2:**
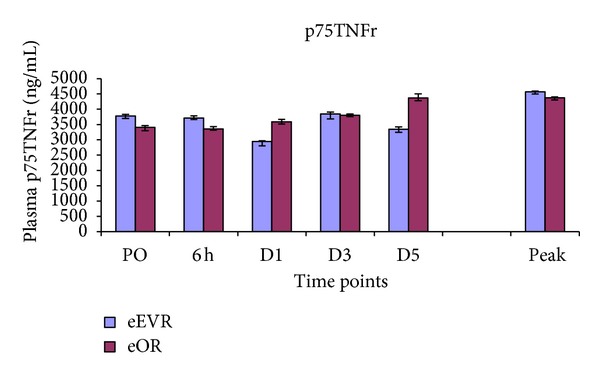
Plasma p75TNFr concentrations expressed as median and interquartile range. At D5 *P* = 0.04 eEVR versus eOR; D5 versus PO in eOR *P* = 0.02.

There was no change in the plasma p75TNFr in the eEVAR group postoperatively at any time points compared to preoperative (PO) level (Figure 2). However, the eOR group showed an increase in plasma p75TNFr from D3 (*P* = 0.06), reaching significance on D5 (*P* = 0.02). Plasma p75TNFr concentration was the same in both groups except on D5 [3344 (2356–4093 pg/mL) versus 4368 (3249–4663 pg/mL) (*P* = 0.04)] (Figure 2).

### 3.4. Neutrophil Elastase/*α*1-Antitrypsin Complex (NE/AAT)

**Figure 3:**
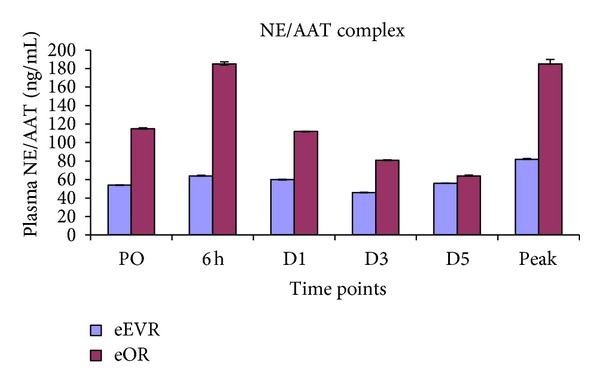
Plasma NE/AAT concentrations expressed as median and interquartile range. At PO, 6 h, D1 and peak *P* < 0.05.

The peak postoperative plasma NE/AAT concentrations were higher in the eOR group (185 (109–689 ng/mL)) compared to the eEVAR group (82 (61–163 ng/mL) (*P* = 0.01)) (Figure 3). Greater NE/AAT concentrations were observed in the eOR group compared to the eEVAR group at PO (115 (71–196 ng/mL) versus 54 (33–85 ng/mL) (*P* = 0.01)), 6 h (185 (70–308 ng/mL) versus 64.0 (50–121 ng/mL) (*P* = 0.02)) and D1 (112 (63–275 ng/mL) versus 60 (21–95 ng/mL) (*P* = 0.02)). However, this significance was lost by D3 (81 ng/mL versus 46 ng/mL (*P* = 0.09)) and D5 (64 ng/mL versus 56 ng/mL (*P* = 0.854)) (Figure 3). Elevations of borderline significance in NE/AAT were observed at 6 h (185 (70–300 ng/mL), *P* = 0.06) and D3 (81 (46–124) *P* = 0.05) compared to PO (115 (71–196 ng/mL)) in the eOR group. The other comparisons with baseline in both groups failed to reach significance.

### 3.5. C-Reactive Protein (CRP)

**Figure 4:**
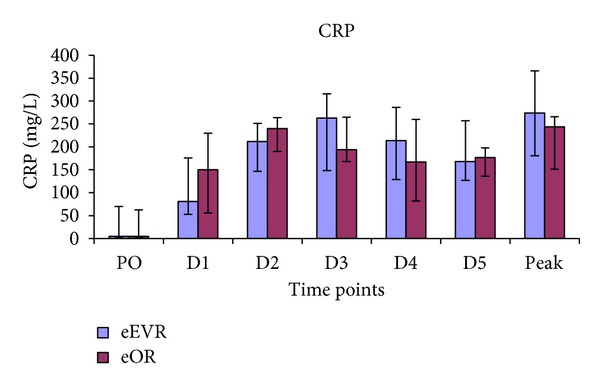
Serum CRP concentrations expressed as median and interquartile range.

A significant rise in serum CRP was observed in both groups postoperatively, peaking on D2 in the eOR group and a day later in the eEVAR group (Figure 4 and Table 1). However, there was no significant difference between the groups at any of the time points. Similarly, no significant difference was found in the peak CRP between the two groups (Figure 4).

### 3.6. Oxidative Stress

#### 3.6.1. Lipid Hydroperoxide (LOOH)

**Figure 5:**
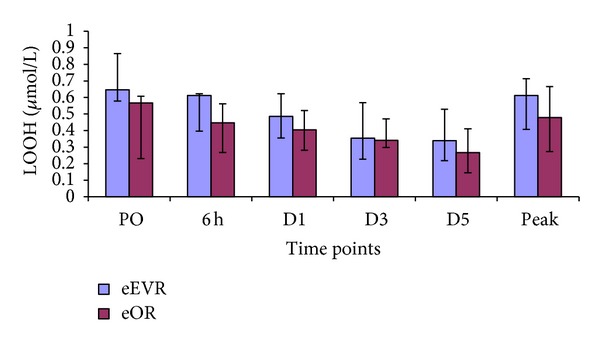
Serum LOOH in the eEVR versus eOR groups at PO (*P* = 0.098), 6 h (*P* = 0.325), D1 (*P* = 0.349), D3 (*P* = 0.896), D5 (*P* = 0.678), and the peak (*P* = 0.56) expressed as median, and IQR.

Even though there was no difference between the groups at peak concentration or any of the individual time points, a significant decrease in serum LOOH was demonstrated at all the postoperative time points compared to the preoperative levels within the groups (Figure 5 and Table 2).

### 3.7. Antioxidant

#### 3.7.1. Vitamin C

**Figure 6:**
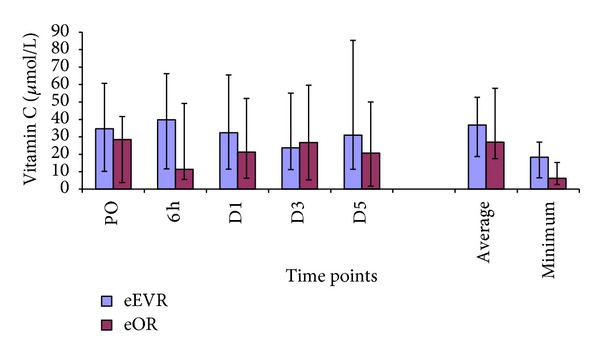
Plasma vitamin C levels expressed as median and IQR.

There was no difference between or within the groups in the vitamin C concentrations (Figure 6; *P* > 0.05). A negative correlation was observed between the average & minimum vitamin C and the duration of surgery and also between average vitamin C and the preoperative systolic blood pressure.

#### 3.7.2. Vitamin E (*α*-Tocopherol)

**Figure 7:**
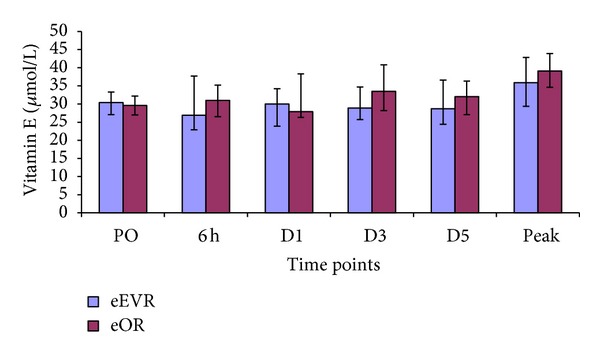
Serum vitamin E/lipid ratio (*μ*mol/L–mmoL/L) expressed as median and IQR.

There was no significant difference between the two groups in serum levels of vitamin E/lipid ratio at any of the studied times, average or the minimum (Figure 7). There were also no significant changes within the groups at any of the postoperative time points compared to preoperative levels (*P* > 0.05).

#### 3.7.3. *β*-Carotene

**Figure 8:**
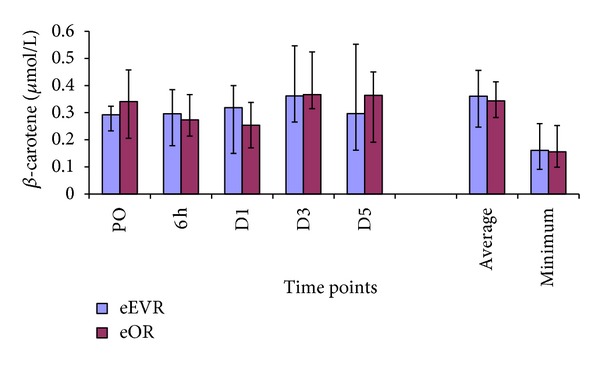
Serum *β*-carotene levels expressed as median and IQR.

There was no significant difference in serum *β*-carotene concentrations between the two groups at any of the time points, average or minimum (Figure 8). A borderline significant increase in serum *β*-carotene levels was found at D3 compared to PO (*P* = 0.02) within the eEVAR group. However, no significant difference in serum *β*-carotene was observed at any of the other time points in this group. Within the eOR group, no significant change in serum *β*-carotene level was demonstrated at any of the postoperative time points compared to PO level.

### 3.8. Lycopene

**Figure 9:**
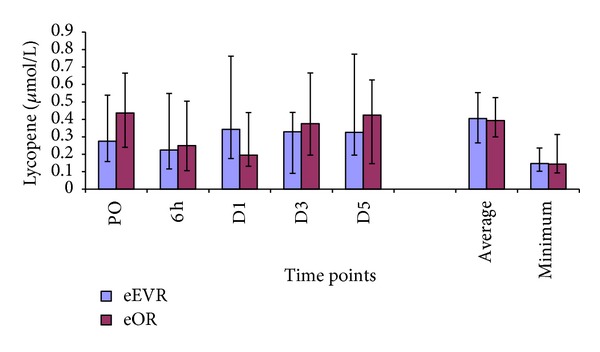
Serum lycopene levels expressed as median and IQR.

There was no significant difference in serum lycopene concentrations between the eEVAR and the eOR groups at any of the measured time points, the postoperative average or the minimum (Figure 9). However, there was a significant decrease in serum lycopene concentrations at D1 compared to PO levels (*P* = 0.02) in the eOR group. Within the eEVAR group, no significant change in serum lycopene level was demonstrated at any of the postoperative time points compared to PO level.

## 4. Discussion

### 4.1. Cytokine Generation

The IL-6 elevation preoperatively in both groups reflects the impact of the global hit of haemorrhagic shock, contrary to the IL-6 peak reported at 24 hours postoperatively in open rAAA repair patient [17]. This discrepancy may be the tertiary referrals in the present study. The travelling time involved in referral to a tertiary centre may be responsible for the very elevated preoperative IL-6 concentrations.

In the eOR group the IL-6 was highest at D1, suggesting a second hit of open AAA repair, similar to elective patients [18]. The fact that IL-6 in the eEVAR group steadily decreased from preoperative levels to reach its lowest level by D5 and the higher peak level in the eOR group (*P* = 0.04) is suggestive of more tissue injury in relation to open surgery [19]. The difference in IL-6 response is related to other factors, such as the intestinal manipulation, exposure of the abdominal viscera, mesenteric traction, and lower limb ischemia-reperfusion injury in the eOR group [20]. The peak of IL-6 at 24 hours occurs also in elective repair, with less in EVAR [21]. Other factors influencing IL-6 include blood loss, blood transfusion and duration of surgery [22].

### 4.2. p75TNFr

TNF-*α* has been variably detected following aortic surgery [22, 23]. TNF-*α* assays can be difficult and as it detected in the plasma when it overflows from tissue production [24]. Therefore, soluble TNF receptors are better markers of TNF activity and are correlated with disease activity in inflammatory conditions and mortality [25].

The increase in p75TNFr in the eOR group could be because of a slow release of p75TNF [18, 26]. At day 5 p75TNFr was higher in the eOR group suggesting a greater ischaemia-reperfusion injury following OR compared to EVAR, superimposed on the initial insult. The higher levels are also due to hemorrhagic shock, lower torso ischaemia and reperfusion, and bowel manipulation [19, 25].

### 4.3. Neutrophil Activation and CRP

Lau et al. found an increased concentration of NE/AAT complex in transperitoneal repair of AAA postoperatively, compared to extraperitoneal approach [11]. This may be due to the bowel manipulation and traction in the transperitoneal group. The initial shock appeared to prime patients' immune systems as shown by the high preoperative NE/AAT levels. The eOR group was exposed to bowel manipulation, mesenteric traction, and ischaemia-reperfusion of the bowel and lower torso, thereby further elevating the neutrophil activation. The higher NE/AAT concentration has been shown to be in those with gut mucosal hypoperfusion [21].

Magnotti et al. demonstrated that when mesenteric lymph was diverted after shock but before gut reperfusion, lung damage was partially prevented [27]. Therefore, gut ischaemia-reperfusion produces inflammatory mediators that produce distant organ damage by neutrophil recruitment [28]. Neutrophil activation produces local inflammation resulting in increased bowel permeability. Gut ischaemia-reperfusion increases cytokine release and impaired intestinal barrier function and bacterial translocation [29]. CRP correlates with the acute inflammation [30]. In the present study both groups had increased CRP postoperatively, peaking at days 2 and 3. Others have observed no difference in CRP between elective EVAR and OR, peaking at day 3 [31].

### 4.4. Oxidative Stress and Antioxidants

Lipid hydroperoxides (LOOH) are a generic biomarker for oxidative stress status [32]. Both groups had similar serum LOOH postoperatively. This finding contradicts our hypothesis that eEVAR could cause lower oxidative stress than eOR. LOOH was highest preoperatively reflecting the impact of body ischaemia. The collapsed stage represents decompensation followed by physiological body compensatory mechanisms. The high LOOH preoperatively suggests that the oxidative stress due to total body ischaemia-reperfusion injury exceeds in magnitude the insult of surgical intervention and ischaemia-reperfusion injury. The lack of correlation between LOOH and the perioperative variables could be because of interrelationship between these variables in addition to the impact of stress related to delay between the initial symptoms and the surgical intervention. The consumption of vitamin E during lipid peroxidation induced by oxygen radicals in ischaemia-reperfusion prevents the development of tissue damage [33]. Vitamin E supplementation does reduce skeletal muscle damage during ischaemia-reperfusion [34].

In the present study, no intergroup difference was observed in antioxidant vitamins, suggesting similar vitamin consumption. However, there was a significant negative correlation between the serum vitamin C level and the duration of surgery, due to increased intraoperative vitamin C consumption. However, while the latter was not supported by the correlation between the LOOH and duration of surgery, it could be because of the high LOOH generated by the initial insult from the hemorrhagic shock.

In summary, the aim of this study was to compare the inflammatory response after endovascular and open aneurysm repair. The main results showed that the elevated inflammatory cytokines preoperatively rose further after repair, especially if preformed as an open procedure. The oxidative stress was most marked preoperatively, reflecting a greater insult from the rupture than the repair. In conclusion, EVAR provokes a lesser systemic inflammatory response compared to OR, and future research should target its modulation.

## Figures and Tables

**Table 1:** Within-group comparison of CRP concentrations.

	6H-PO	D1-PO	D3-PO	D5-PO
eEVR	0.05	0.02	0.003	0.005
eOR	0.005	0.003	0.001	0.002

**Table 2:** Within-group comparison of LOOH.

	PO-D1	PO-D2	PO-D3	PO-D4	PO-D5
eEVR (p)	0.001	0.002	0.003	0.001	0.002
eOR (p)	0.001	0.001	0.002	0.005	0.006
